# External validation of a microRNA thyroid classifier: a real-world prospective study

**DOI:** 10.1530/ETJ-25-0105

**Published:** 2025-12-18

**Authors:** Eduarda Gregorio Arnaut Lima, Kely Silveira Marcello, Acklei Viana, Daniel Knabben Ortellado, Gustavo Philippi de Los Santos, Jalmir Rogério Aust, Alvin Laemmel, André Wüst Zibetti, Maria Isabel Cunha Vieira Cordioli

**Affiliations:** ^1^ Division of Endocrinology, Department of Internal Medicine, Federal University of Santa Catarina, Florianópolis, Santa Catarina, Brazil; ^2^ Integrated Center for Head and Neck Surgery (NICAP), Florianópolis, Santa Catarina, Brazil; ^3^ Otorhinolaryngological Diagnostic Center (CDO), Florianópolis, Santa Catarina, Brazil; ^4^ Department of Informatics and Statistics, Federal University of Santa Catarina, Florianópolis, Santa Catarina, Brazil

**Keywords:** thyroid nodule, thyroid cancer, thyroid molecular test, microRNA

## Abstract

**Introduction:**

Thyroid nodules are common, affecting approximately 50% of individuals. These nodules are often discovered incidentally and exhibit benign characteristics. Following a suspicious ultrasound, a fine-needle aspiration biopsy (FNAB) is performed to assess the risk of malignancy. However, approximately 30% of cases are classified as indeterminate by cytology. In response, the development of molecular tests has refined malignancy risk assessment and reduced the need for diagnostic surgeries.

**Objective:**

To independently evaluate the real-world clinical utility and the diagnostic performance of a microRNA-based molecular test (mir-THYpe full) in improving diagnostic accuracy and avoiding unnecessary surgeries in indeterminate thyroid nodules.

**Methods:**

This is the first external, independent, prospective, real-world, observational, and non-interventional validation study of this molecular classifier. A total of 256 patients with nodules classified as Bethesda III/IV were analyzed.

**Results:**

The test was positive for malignancy in 90 patients, 79 (90%) of whom underwent surgery. Of the 158 test-negative nodules, 7 (4.4%) underwent thyroidectomy. The test demonstrated a sensitivity of 83.0%, a specificity of 83.5%, a positive predictive value of 62.8%, and a negative predictive value of 93.6%.

**Conclusions:**

The mir-THYpe full molecular test supported 95.5% of clinical decisions when negative and 89.8% when positive, reducing surgery rates by 79.5%. Therefore, the integration of this microRNA-based classifier into clinical practice represents a valuable tool in managing indeterminate thyroid nodules, reducing unnecessary thyroidectomies, and conserving valuable healthcare resources.

## Introduction

Thyroid nodules are a common condition that affects approximately 50% of individuals, according to autopsy studies (1). Often discovered incidentally through routine ultrasonography (US), most of these nodules are benign (2). Fine-needle aspiration biopsy (FNAB) is the most accurate, reliable, and cost-effective method for evaluating thyroid nodules with suspicious sonographic features (3, 4). However, approximately 30% of nodules yield indeterminate cytology results, rendering them unclassifiable as either benign or malignant (1). In response to this diagnostic challenge, molecular testing has emerged as a valuable tool to refine risk assessment and reduce the need for diagnostic thyroid surgery (5, 6), being recommended as an option for indeterminate nodules by major clinical guidelines, such as those of the European Thyroid Association (ETA) (7) and the American Thyroid Association (ATA) (1).

Molecular tests have been refined over the years based on knowledge gained about the pathogenesis and biomarkers of tumors (8). Early molecular tests relied solely on the somatic mutations within tumors and had a low sensitivity. Nevertheless, the development of new tests capable of detecting a broader range of molecular alterations – such as point mutations, copy number alterations, gene fusions, and gene and microRNA expression – has significantly improved the specificity and sensitivity of molecular testing (9).

In countries outside the USA, the mir-THYpe full molecular test is the most widely used thyroid molecular test for the investigation of indeterminate nodules in clinical practice (10, 11). In the first validation study, focused on the diagnostic performance, the mir-THYpe full test reached 94.6% sensitivity, 81% specificity, 95.9% negative predictive value (NPV), and 76.1% positive predictive value (10). Subsequently, a second validation study was published, focusing on the real-world clinical utility of the test. The authors reported a 74.6% reduction in potentially unnecessary surgeries in a prospective, multicenter cohort of 435 patients (11). Although both studies were well designed and published in high-impact, peer-reviewed journals, neither study is entirely independent due to the involvement of in-house researchers from Onkos, the company that developed the test.

This study represents the first entirely external and independent validation of the mir-THYpe full molecular test, evaluating a real-world, multicenter, and prospective cohort of patients. It aims to assess the test’s diagnostic performance and clinical utility in refining diagnosis and guiding treatment decisions for patients with indeterminate thyroid nodules.

## Methods

### Study design and patient data collection

This independent, prospective, multicenter, observational, and non-interventional study analyzed patients who underwent the mir-THYpe full molecular test – a microRNA-based molecular test (see details in Supplementary Table 1 (see section on Supplementary materials given at the end of the article)) – as part of real-world clinical routine between February 2018 and December 2021 (when the follow-up period finished) in three different sites in the state of Santa Catarina, Brazil. At the time the study was completed, the assay did not include concomitant analysis of somatic mutations (e.g. BRAF, TERT, RAS) in addition to the microRNA profile. Unfortunately, to date, the mir-THYpe full molecular test is not yet available in the public health system in Brazil. Therefore, all patients included in this study were being followed up in the private health system.

The study population consisted of individuals aged ≥18 years with thyroid nodules who underwent FNAB procedures, with real-world cytopathology analysis classifying the samples as Bethesda classes III or IV (12, 13), and who had a signed medical prescription for the use of the mir-THYpe full and privately paid for the molecular test. No clinical or other criteria were imposed on the physicians to select patients for the molecular testing. This was based on a shared decision between the attending physician and the patient. Samples were included prospectively and consecutively, ensuring representative inclusion of the target population, minimizing selection bias, and enhancing the external validity of the findings. The mir-THYpe full molecular test was performed using the same FNAB cytology slides classified as Bethesda III or IV by the pathologists, eliminating the need for additional or fresh FNAB sampling. Molecular testing results were obtained from the official final reports provided to patients, indicating either a positive or a negative result for malignancy. Among patients with negative mir-THYpe results who did not undergo surgery during the study period, the follow-up time was defined as the interval from the date the result was issued to 31 December 2021 (mean: 706 days; range: 10–1,451 days). For patients who underwent thyroidectomy, histopathological (HP) reports served as the reference standard (gold standard). Histopathological analysis of surgical specimens was performed by local pathology services as part of routine clinical care. No centralized blinding protocol was applied, and it was not possible to determine whether pathologists had access to the mir-THYpe test results before diagnosis. This approach reflects standard diagnostic workflows in real-world settings, where molecular findings – when available – may complement histological interpretation. Nodule-level correspondence was determined based on side (left, right, or isthmus), segment (upper, lower, or central), and size features documented in the FNA report, and these were matched to the gross and HP reports. Cases in which correspondence could not be confidently established were excluded from the analyses. Collision tumors comprising benign and malignant components within the same nodule were rare and, when identified, were also excluded from the analysis. The HP reports were provided either by the participating physicians or directly by the pathology laboratories.

All analyses were performed at the nodule level. In cases where a patient had more than one nodule tested, each nodule was considered independently, based on its specific cytological classification, molecular test result, and available histopathological outcome.

Despite the benign or indolent nature of non-invasive follicular thyroid neoplasms with papillary-like nuclear features (NIFTPs), their diagnosis can only be confirmed after applying strict histological criteria following surgery. Therefore, NIFTPs were included in the ‘malignant’ group, and positive test results were regarded as true positives (1).

### Ethical approval

The study protocol was approved by the Ethics Committee of Research in Human Beings at the Federal University of Santa Catarina (CEPSH-UFSC) and listed under registration number 5.576.696. Written informed consent was obtained from all participants.

### Statistical analysis

Statistical analyses were conducted using R software (Austria), an open-source statistical programming environment (R Core Team, 2022). Confidence intervals were calculated using the Clopper–Pearson ‘exact’ method. Associations between variables were tested using the Pearson Chi-Square test and the Fisher ‘exact’ test. The NPV for cases with negative test results and no surgical treatment was calculated using Bayes’ Theorem, based on Hall’s work (14).

## Results

### Patient and nodule characteristics

**Figure 1:**
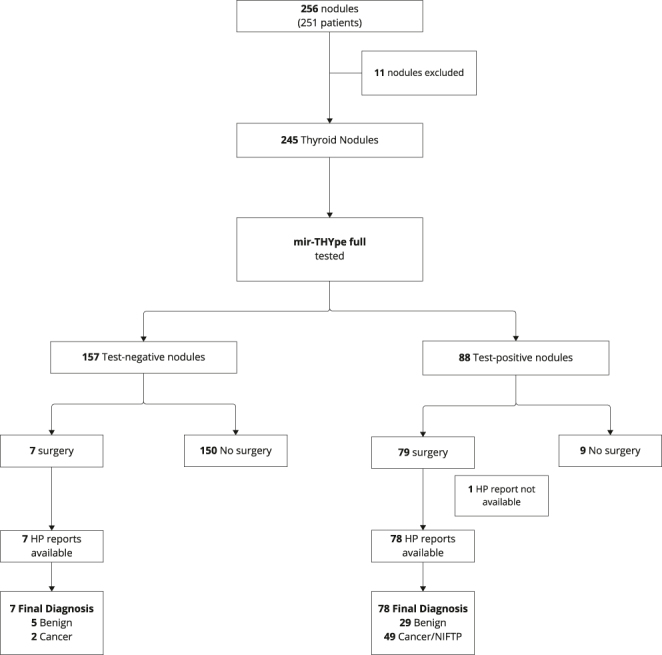
Flowchart of the study evaluating thyroid nodules using the mir-THYpe full molecular test.

A total of 251 patients with 256 nodules with indeterminate cytology who were tested with the mir-THYpe full molecular test were included in this study. Of these, 11 nodules (4.3%) were excluded due to the presence of both benign and malignant lesions in the same thyroid region, as reported in the HP analysis. The remaining 245 nodules classified as Bethesda III and IV, and fulfilling all initial inclusion criteria, were categorized according to the mir-THYpe full molecular test results (negative or positive for malignancy). Of these, 157 (64.1%) nodules were classified as test-negative and 88 (35.9%) nodules as test-positive. Among the test-negative nodules, seven underwent surgery with histopathological analysis, confirming five benign cases and identifying two malignant lesions. In the test-positive group, 79 nodules underwent surgical intervention. Follow-up was not available for one case, while 78 had accessible pathology reports. These cases resulted in a final diagnosis of 29 benign lesions – the majority being follicular adenomas, a known challenge in thyroid cytopathology – and 49 cancer/NIFTP lesions (Fig. 1).

The mean age of the patients was 51.6 years, and the majority, 205 (83.7%), were female. A total of 191 (78%) nodules were classified as Bethesda IV based on the FNAB results, while 54 samples (22%) were classified as Bethesda III (Table 1). The mean nodule size was 1.2 cm. In summary, the ‘average patient profile’ of a real-world patient referred for the mir-THYpe full molecular test in this study was a woman (83.6%) with Bethesda IV cytology (73%) and a thyroid nodule <1.98 cm.

**Table 1:** Clinical characteristics of the study cohort. Data are presented as *n* (%).

Variable	Overall	mir-THYpe full results		*P*-value*
		Negative	Positive	
Nodules	245 (100.0)	157 (64.1)	88 (35.9)	<0.001
Age				0.04
20–54	139 (56.7)	81 (51.6)	58 (65.9)	
>54–90	106 (44.3)	76 (48.4)	30 (34.1)	
Sex				0.12
Female	205 (83.7)	127 (80.9)	78 (88.6)	
Male	40 (16.3)	30 (19.1)	10 (11.4)	
Bethesda class				>0.9
III	54 (22.0)	35 (22.3)	19 (21.6)	
IV	191 (78.0)	122 (77.7)	69 (78.4)	
Surgery				<0.001
No	159 (64.9)	150 (95.5)	9 (10.2)	
Yes	86 (35.1)	7 (4.5)	79 (89.8)	

* Pearson’s Chi-squared test; Fisher’s exact test.

### Test results and performance

The mir-THYpe full molecular test was positive for malignancy in 88 nodules (malignant call rate: 88/245; 35.9%), of which 79 nodules (89.8%) underwent surgical treatment during the study follow-up period. Of the 157 nodules with negative results (benign call rate: 157/245; 64.1%), 150 nodules were not treated with surgery (150/157; 95.5%) (Table 1). Of the seven patients with negative mir-THYpe full molecular test results who did have surgery, five (5/7; 71.4%) were confirmed to be benign lesions in the HP reports, while two (2/7; 28.6%) were diagnosed as malignant nodules. Interestingly, these two false-negative cases were submitted to surgery due to another indeterminate nodule, which was classified as positive in the mir-THYpe test (both true positives). Of the 79 resected nodules with positive results from the molecular test, 49 nodules (49/79; 62%) were confirmed as cancer, including 2 (2/49; 4.1%) identified as NIFTP lesions (true positives) in the postsurgical HP report (Table 2). All the samples with false negative/positive results are described in Supplementary Table 2.

**Table 2:** Performance of the mir-THYpe full test according to histopathological tissues.

Postsurgical histologies	Total, *n* (%)
mir-THYpe full negative results	7
Benign nodules – true negative	5 (71.4)
Follicular adenoma	4
Oncocytic adenoma of the thyroid	1
Malignant nodules – false negative	2 (28.6)
Papillary thyroid micro/carcinoma variant follicular	1
Follicular thyroid carcinoma minimally invasive	1
mir-THYpe full positive results	78
Benign nodules – false positive	29 (37.2)
Follicular adenoma	21
Thyroid follicular nodular disease	5
Oncocytic adenoma of the thyroid	1
Thyroiditis	2
Malignant nodules – true positive	49 (62.8)
Papillary thyroid micro/carcinoma variant follicular	38
Follicular thyroid carcinoma minimally invasive	4
Papillary thyroid micro/carcinoma classic	2
Papillary thyroid micro/carcinoma subtype solid	1
Papillary thyroid microcarcinoma subtype oncocytic	1
Oncocytic microcarcinoma of the thyroid	1
NIFTP	2
Total	85

To minimize the effect of the small number of resected test-negative nodules on the calculation of sensitivity and NPV, and since it could not be assumed that all the other test-negative nodules were truly benign, a theoretical calculation based on Bayes’ theorem was performed (14). The sensitivity observed during the validation study (94.6%) was applied to the 150 non-resected nodules with test-negative results, resulting in 142 theoretical true benign and 8 malignant nodules (10). In this context, the mir-THYpe full molecular test performed a sensitivity of 83.0% (95% CI: 71.0–91.6) and a specificity of 83.5% (95% CI: 77.2–88.7). The PPV observed was 62.8% (95% CI: 54.3–70.6), and the NPV was 93.6% (95% CI: 89.3–96.3) at a 25.1% (95% CI: 19.7–31.2) disease prevalence. The test accuracy was 83.4% (95% CI: 78.0–87.9) based on the cases with final histological diagnosis (Table 3).

**Table 3:** Calculated performance of the mir-THYpe full test in the study cohort using Bayes’ theorem.

	Positive	Negative	%	95% CI
Postsurgical tissue class, *n* (%)				
Cancer + NIFTP	49	29		
Benign	8* + 2 (10*)	142* + 5 (147*)		
Test performance				
Sensitivity			83.0	71.0–91.6
Specificity			83.5	77.2–88.7
NPV			93.6	89.3–96.3
PPV			62.8	54.3–70.6
Accuracy			83.4	78.0–87.9

* Theoretical values, considering the published sensitivity (13) of 94.6% for the 150 test-negative nodules not surgically resected.

CI, confidence interval; NPV/PPV, negative/positive predictive value; NIFTP, non-invasive follicular thyroid neoplasm with papillary-like nuclear features.

### Support to clinical decisions and surgery reduction rates

Within the follow-up period, the mir-THYpe full molecular test guided initial therapeutic decisions in 95.5% of cases with a negative result and in 89.8% of cases with a positive result. Overall, the test results aligned with physician management decisions in 93.5% of nodules. It is important to note that most test-negative nodules were not operated on but may remain under clinical surveillance (e.g., ultrasound or repeat cytology), as is standard practice.

To calculate the rate of surgeries avoided among the 245 patients included in the final analysis, it was assumed that 236 would have undergone thyroidectomy if no molecular tests had been available. This assumption excludes nine patients who did not undergo surgery despite having a positive result (Fig. 1). Of these, 150 patients with negative results avoided surgery, representing 63.6% (95% CI: 57.2–69.4) (150/236) of all potential surgeries.

Applying Bayes’ theorem, it is estimated that, among the 150 nodules with a negative molecular test that did not undergo surgical treatment, 142 would be true negative lesions (Table 3). Thus, a total of 176 patients would have been subjected to a ‘potentially unnecessary’ surgery if molecular testing had not been available (142 true negatives +5 with both test-negative and biopsy-negative results +29 with test-positive but biopsy-negative results). Considering that only 36 patients (29 with test-positive and final benign diagnosis +5 with test-negative and final benign diagnosis) were surgically treated, the mir-THYpe full molecular test avoided 79.5% (95% CI: 73.0, 84.8) of ‘potentially unnecessary’ surgeries.

## Discussion

This study represents the first external and independent real-world evaluation of the mir-THYpe full molecular test’s performance in a population of adult patients with indeterminate thyroid nodules. Although the mir-THYpe full molecular test has been widely used in Brazil and, more recently, in several other countries, the absence of an external and independent validation study – conducted without the involvement of the company that developed the test (Onkos Molecular Diagnostics, Brazil) – has been a significant gap in the literature, limiting its ability to fully support clinical decision-making and hindering its incorporation into healthcare systems.

A total of 256 thyroid nodules classified as Bethesda III or IV and tested with the mir-THYpe full molecular test were analyzed. Due to the real-world and multicenter nature of this study, and since these patients were followed in different healthcare facilities, it was not possible to standardize the indication criteria among physicians for molecular testing. Despite the FNAB cytology smear slides used for the molecular test being prepared in various pathology laboratories, employing different fixation and staining protocols, all FNAB samples were successfully analyzed and subjected to molecular analysis.

In the present study, the PPV (62.8%) was slightly lower than that reported in the initial validation study of the mir-THYpe full molecular test (76.1%) (10), but comparable to that observed in the subsequent real-world study (66.2%) (Table 4) (11). For a thyroid molecular test to be considered effective in predicting malignancy (‘rule-in’ test), it must have a high PPV. According to Vargas *et al.* 2018 study, the minimum PPV required for a thyroid molecular test to be deemed effective is a specificity rate above 80%, which would generally result in a PPV exceeding 60% for a condition with a prevalence rate greater than 25% (15). Consequently, the mir-THYpe full molecular test qualifies as a rule-in test based on our findings.

**Table 4:** Performance of the microRNA-based molecular classifier and comparison among previous studies. Data are presented as % (95% CI).

Test performance	Santos *et al.* (10)	Santos *et al.* (11)	This study
Sensitivity	94.6 (81.8–99.3)	89.3 (82.0–94.3)	83.0 (71.0–91.6)
Specificity	81.0 (68.6–90.1)	81.65 (76.6–86.0)	83.5 (77.2–88.7)
PPV	76.1 (65.0–84.5)	66.2 (60.3–71.7)	62.8 (54.3–70.6)
NPV	95.9 (85.9–98.9)	95* (91.7–97.0)	93.6* (89.3–96.3)
Accuracy	86.3 (77.7–92.5)	83.8 (79.8–87.4)	83.4 (78.0–87.9)
Disease prevalence	38.9 (29.1–49.5)	28.7 (24.3–33.5)	25.1 (19.7–31.2)
Clinical decisions supported	Not evaluated	92.3%	93.5%
Total avoided surgeries	Not evaluated	52.5 (47.6–57.3)	63.6 (57.2–69.4)
Potentially avoided surgeries	Not evaluated	74.6 (69–79.2)	79.5 (73.0–84.8)

CI, confidence interval; PPV, positive predictive value; NPV, negative predictive value.

* NPV based on the sensitivity and Bayes’ theorem.

The majority of nodules with a negative result on the mir-THYpe full molecular test (150/157; 95.54%) were not subjected to surgery. Therefore, due to the limited number of test-negative nodules treated surgically in real-world settings, the NPV was estimated using Bayes’ theorem, as described in the Methods section, resulting in a calculated NPV of 93.6%. When a diagnostic test is designed to predict benign nodules (a ‘rule-out’ test), it requires a high NPV. Statistically, a thyroid molecular test should achieve an NPV of at least 94% (15), with a residual malignancy risk of less than 6% for a negative result, which is comparable to a Bethesda II cytology. Therefore, the mir-THYpe full molecular test can also be considered a rule-out test based on our findings.

The two major commercially available molecular tests for indeterminate thyroid nodules are Veracyte´s Afirma Genomic Sequencing Classifier (GSC) and CBLPath’s ThyroSeq v3 (16). The Afirma GSC utilizes RNA next generation sequencing (NGS) combined with data-based machine learning analysis. It is considered a ‘rule-out’ test, as its validation study demonstrated a high NPV of 96%, but a low PPV of 47%. The ThyroSeq v3, on the other hand, is a DNA- and RNA-targeted NGS platform, with results reported as positive or negative, along with detailed molecular findings, risk of recurrence, and suggested personalized patient management. It is classified as both a ‘rule-out’ and ‘rule-in’ test, with an NPV of 97.6% and a PPV of 66% (17). Although well-established and well-validated, thyroid molecular tests such as Afirma and ThyroSeq v3 face significant cost-related challenges outside the USA. In Latin America, Africa, Europe, and Asia, the mir-THYpe full molecular test has an average cost that is 3–5 times lower, making it a more affordable option (18).

Thyroid surgery is generally safe, but potential complications include postoperative bleeding, injury to the recurrent laryngeal nerve, and permanent hypoparathyroidism (1). In this study, within the follow-up period, the mir-THYpe full molecular test supported 95.5% of clinical decisions when the test was negative for malignancy and 89.8% when the test was positive, resulting in a surgery avoidance rate of 63.6%.

This study has some limitations. A significant number of patients with a negative result did not undergo surgery, and structured clinical follow-up data (e.g., ultrasound surveillance) were not uniformly available. In a real-world, multicenter setting, particularly when patients receive a benign molecular result, it is common for follow-up to be discontinued by the patient or performed at other institutions, making standardized data collection challenging. Despite these constraints, the use of Bayes’ theorem allows a theoretical estimation of test performance in non-operated cases. In addition, due to the slow and typically indolent nature of malignant thyroid nodules, identifying any missed cancer would require a long time frame beyond the prospective duration of this study, making long-term follow-up a challenge. However, this is a common challenge in observational real-world studies due to ethical concerns regarding unnecessary surgeries. While focusing only on patients who underwent surgery could address this issue, it may introduce another bias, as those who had surgery despite a negative test are more likely to have a higher malignancy rate than those whose nodules were not surgically removed (19).

The integration of a microRNA-based molecular classifier into clinical practice, as part of a multimodal diagnostic strategy that combines ultrasound features, cytopathology, and clinical risk factors, has emerged as a valuable tool for guiding the management of thyroid nodules with indeterminate cytology. This approach significantly reduces the need for thyroidectomies and, consequently, conserves valuable healthcare resources. Based on the findings of the present study, along with the results from the previous validation studies of the molecular test, the data support the use of the mir-THYpe full molecular test in clinical practice to facilitate a more accurate pre-surgical diagnosis and informed decision-making in the management of patients with indeterminate thyroid nodules.

## Supplementary materials





## Declaration of interest

The authors declare that there is no conflict of interest that could be perceived as prejudicing the impartiality of the research reported. None of the authors have a financial or institutional conflict of interest with Onkos Molecular Diagnostics, the developer of the test evaluated in this study.

## Funding

This research did not receive any specific grant from any funding agency in the public, commercial, or non-profit sector.

## Author contribution statement

EGAL conceived the study (together with MICVC and KSM) and wrote the paper. EGAL and KSM made the database. EGAL performed data collection. EGAL, MICVC, KSM, and AWZ made the statistical analysis. AV, DKO, GPLS, JRA, AL, and other authors have taken part in data management and have read, commented on, and accepted the final manuscript.
