# Supplementary material for: External validation of a microRNA thyroid classifier: a real-world prospective study
Source: Eur Thyroid J. 2025 Dec 18;14(6):e250105. doi: 10.1530/ETJ-25-0105 (PMC12720197; doi:10.1530/ETJ-25-0105)
Supplement: Supplementary file 1 [file supplementary_table_1.pdf]

Supplementary Table 1. List of microRNAs used in the mir-THYpe full panel

**Normalizers**	**Discriminators**
let-7a	miR-146b
miR-103	miR-152
miR-125a-5p	miR-155
let-7b	miR-200b
miR-145	miR-181b
RNU48	

As reported by Santos MT et al., 2018 (10)

Santos MTD, Buzolin AL, Gama RR, Silva ECAD, Dufloth RM, Figueiredo DLA, Carvalho AL. Molecular Classification of Thyroid Nodules with Indeterminate Cytology: Development and Validation of a Highly Sensitive and Specific New miRNA-Based Classifier Test Using Fine-Needle Aspiration Smear Slides. Thyroid. 2018 Dec 1;28(12):1618–26
