# Supplementary material for: External validation of a microRNA thyroid classifier: a real-world prospective study
Source: Eur Thyroid J. 2025 Dec 18;14(6):e250105. doi: 10.1530/ETJ-25-0105 (PMC12720197; doi:10.1530/ETJ-25-0105)
Supplement: Supplementary file 2 [file supplementary_table_2.pdf]

Supplementary Table 2. Demographic, clinical, molecular and pathological characteristics of cases with false negative or false positive test result

Patient ID	Age, y	Sex	Nodule size (cm)	Bethesda class	Postsurgical histologies
**False Negatives**					
89	43.5	F	-	IV	Papillary thyroid micro/carcinoma variant follicular
91	57.5	F	1.2	IV	Follicular thyroid carcinoma minimally invasive
**False Positives**					
11	40.2	F	0.7	III	Follicular adenoma
22	41.4	F	5	III	Follicular adenoma
55	59.3	F	0.9	III	Follicular adenoma
73	55.4	F	-	III	Follicular adenoma
80	75.1	F	-	III	Follicular adenoma
6	41.1	F	3.6	III	Oncocytic adenoma of the thyroid
34	82.0	F	2.1	III	Thyroid follicular nodular disease
37	42.5	F	1.3	III	Thyroiditis
9	52.2	M	0.9	IV	Follicular adenoma
15	35.9	F	2	IV	Follicular adenoma
25	61.1	F	2.6	IV	Follicular adenoma
26	-	F	-	IV	Follicular adenoma
31	74.9	F	-	IV	Follicular adenoma
33	67.1	F	1.1	IV	Follicular adenoma
41	33.6	F	-	IV	Follicular adenoma
42	44.8	F	1.7	IV	Follicular adenoma
42	44.8	F	1.7	IV	Follicular adenoma
51	61.8	M	1.3	IV	Follicular adenoma

53	56.3	F	-	IV	Follicular adenoma
62	30.0	F	3	IV	Follicular adenoma
65	54.0	M	1.4	IV	Follicular adenoma
66	61.4	F	1.0	IV	Follicular adenoma
68	43.5	F	-	IV	Follicular adenoma
69	49.3	F	-	IV	Follicular adenoma
21	48.9	M	1.1	IV	Thyroid follicular nodular disease
24	42.3	F	-	IV	Thyroid follicular nodular disease
43	49.1	F	-	IV	Thyroid follicular nodular disease
49	31.3	F	1.2	IV	Thyroid follicular nodular disease
27	38.0	F	0.7	IV	Thyroiditis

y, years; cm, centimeters
